# Cleft Care India Study: A National Audit on Patients with Non-Syndromic Unilateral Cleft Lip and Palate: Surgical Procedures and Outcomes

**DOI:** 10.1177/10556656261436188

**Published:** 2026-04-10

**Authors:** Panchali Batra, Deborah Sybil, Krishnamurthy Bonanthaya, Subodh Kumar Singh, Srinivas Gosla Reddy, Puneet Batra, Suryakant Das, Syed Altaf Hussain, Ravi K Mahajan, Raj Gopal Reddy, Vinay Chugh, Gayatri Moghe, Nandlal Bhojraj, Krishna Shama Rao, K. Chethana, Sukhdeep Singh Kahlon, Kamlesh Singh, Ranjit H Kamble, Shruti Bijapur, Charanya Vijaykumar, H. Hari Kishore Bhat, Sakshi Katyal, Manoj Prathap, Karthika Nambiar, Badri Thiruvenkatachari

**Affiliations:** 1 Department of Orthodontics, Faculty of Dentistry, 231547 Jamia Millia Islamia, New Delhi, India; 2 Department of Oral Surgery, Faculty of Dentistry, 28849 Jamia Millia Islamia, New Delhi, India; 3 CLC, 78767 Smile Train at Bhagwan Mahaveer Jain Hospital, Bangalore, India; 4 Department of Plastic Surgery, G S Memorial Plastic Surgery Hospital, Varanasi, India; 5 Facial Plastic Surgery, Director (GSR Institute of Craniomaxillofacial and Facial Plastic Surgery), Hyderabad, Telangana, India; 6 231547 Manav Rachna International Institute of Research and Studies, Sant Parmanand Hospital, New Delhi, India; 7 Department of Orthodontics, 450492 SCB Govt Dental College, Cuttack, India; 8 Department of Plastic and Reconstructive Surgery, 63564 Sree Balaji Medical College and Hospital, The Cleft and Craniofacial Centre, Chromepet, Chennai, 600044, India; 9 Smile Train Centre, 163985 Amandeep Hospital, Amritsar, India; 10 Department of Craniomaxillofacial and Facial Plastic Surgery, GSR Memorial Institute, Hyderabad, India; 11 Department of Dentistry, 410730 All India Institute of Medical Sciences, Jodhpur, Rajasthan, 342001, India; 12 Pediatric and Preventive Dentistry, Toothbuddies Dental Clinic, Hyderabad, Telangana, India; 13 Department of Pediatric and Preventive Dentistry, JSS Dental College and Hospital, Mysore, Karnataka, India; 14 Craniofacial Centre, Inga Health Foundation, Bangalore, India; 15 Craniofacial Centre, Inga Health Foundation, Bangalore, India; 16 163985 Orthodontics, Amandeep Hospital, Amritsar, India; 17 Department of Orthodontics and Dentofacial Orthopaedics, 76201 Saraswati Dental College and Hospital, Lucknow, UP, India; 18 Cleft Surgery, Smile train project, Lucknow, India; 19 Vivekananda Polytechnic and Institute of Medical Sciences (VPIMS), Lucknow, UP, India; 20 Smile Train Cleft Centre, 29657 Sharad Pawar Dental College & Hospital, Wardha, Maharashtra, India; 21 78767 Smile Train Cleft Headship Centre, Bhagwan Mahaveer Jain Hospital, Bangalore, India; 22 Sree Balaji Medical College and Hospital, 63564 The Cleft and Craniofacial Centre, Chromepet, Chennai, 600044, India; 23 Yenepoya-ABMSS Cleft Project, Centre for Craniofacial Anomalies, Yenepoya (Deemed to be University), Mangalore, India; 24 Department of Oral & Maxillofacial Surgery, Yenepoya Dental College, Mangalore, India; 25 Department of Orthodontics, Faculty of Dentistry, 245562 Jamia Millia Islamia, New Delhi, India; 26 Sree Balaji Dental College and Hospital, Bharath Institute of Higher Education and Research, Pallikaranai, Chennai, India; 27 School of Medical Sciences, Faculty of Biology, Medicine and Health, Coupland Building 3, Univeristy of Manchester, Manchester

**Keywords:** oral health, cleft lip and palate, outcomes, skeletal morphology, surgical technique

## Abstract

**Objective:**

To assess the surgical protocols followed in the treatment of non-syndromic UCLP across Indian cleft centres and identify factors influencing secondary surgical outcomes.

**Design:**

Prospective cross sectional design.

**Setting:**

The study was conducted in 14 sary/tertiary care comprehensive cleft centres.

**Participants:**

The study included 307 patients with non-syndromic UCLP across three age groups (5, 12, and 20 years) from 14 centres nationwide.

**Outcomes:**

Data was collected through a standardized web-based form. Variables recorded included surgeon specialty and experience, surgical sequence, presurgical orthopaedics, primary surgical techniques, and their association with the need for secondary procedures such as lip/nose revision, velopharyngeal surgery, fistula repair, and alveolar bone grafting. Descriptive statistics were generated, and associations between variables were analysed using Fisher's exact test.

**Results:**

Millard's technique was the most commonly used approach for primary lip repair across all age groups. Presurgical orthopedic plates, nasal conformers, and the specific lip repair technique were not significantly associated with the need for lip or nasal revision surgery, although the wide confidence intervals indicate considerable uncertainty in these estimates. Similarly, the type of palatal repair and use of intravelar veloplasty were not significantly associated with fistula formation or the need for secondary velopharyngeal or fistula repair procedures. Across centres, wide variation existed in techniques used, but no one approach showed superiority in reducing secondary surgery needs.

**Conclusion:**

Despite the observed variability in surgical weak associations were found between specific surgical techniques or surgeon-related factors and the need for secondary surgical interventions. The wide confidence intervals highlight the uncertainty of these findings and the need for further national-level audits, underscoring the challenge of establishing best practices in cleft care without robust systems for comprehensive data collection and consolidation.

## Introduction

To evaluate and improve the healthcare services, standardized measurement of outcomes is a fundamental prerequisite.^1^ Recognizing the complex and varied nature of craniofacial anomalies and their associated morbidities, both the American Cleft Palate-Craniofacial Association and the World Health Organization have emphasized the importance of continuous monitoring and standardization of surgical procedures in the management of cleft lip and palate (CLP), a developmental anomaly with a variable prevalence.^2–5^ This approach supports effective intervention and the delivery of evidence-based care, but also contributes to improved patient outcomes.^2,3^

Recognising the limited availability of nationwide audits and the increasing need to align clinical practice with evidence-based interventions in cleft care, two major multicentre initiatives, the Eurocleft project in Europe and the Americleft project in North America, were undertaken in the past two decades.^6,7^ Both initiatives were prompted by the findings of the CSAG study, which led to significant restructuring of cleft care services in the UK. These large-scale audits examined regional variations in the burden of cleft conditions, surgical and orthodontic care pathways, and patient and parent satisfaction relative to deformity severity and treatment outcomes.^6–8^ Their results revealed considerable discrepancies in surgical protocols, underscoring the wide variation in practice patterns even within individual countries.^6–8^

The Asian subcontinent reports the highest prevalence of CLP globally.^4,5^ India, being the most populous country and a developing nation, contributes substantially to this global burden. Despite the high demand of healthcare services, access to quality and affordable surgical care including cleft management, remains limited in many regions of the country.^5,9^ While the Smile Train initiative has significantly transformed the landscape of CLP care in India, offering free surgeries and raising awareness, India lacks a dedicated national level cleft outcomes audit.^10^ This critical gap was addressed with the launch of the Cleft Care India Study (CCIS), a multi-centric initiative aimed at not only systematically collecting and analysing the national burden of non-syndromic unilateral CLP (UCLP), but also at documenting the orthodontic, speech, and surgical care provided to patients across the country.

Previous publications from CCIS have reported the methodology in detail, and evaluated the other outcomes of unilateral CLP patients managed at the participating centres (work submitted for publication consideration).^11^ The present work aims to identify the surgical protocols followed in the management of patients with non-syndromic UCLP, with a focus on exploring the various factors influencing surgical outcomes.

## Method

This study was approved by the Institutional Ethics Committee (approval no. 18/2279/JMI/IEC/2020) on 10 November 2020 at the Data Coordinating Centre and individual ethical committee approval at each of the participating centres. All participants provided written informed consent prior to enrolment in the study. This research was conducted ethically in accordance with the Declaration of Helsinki.

A total of 14 centres from different regions of the country participated with each centre contributing surgical pre-orthodontic data for at least 30 non-syndromic cleft patients. To ensure uniformity across centres, a standardized data collection protocol was developed, and data was prospectively collected via a dedicated web portal. The portal captured information on the educational background and clinical experience of the operating surgeon. Detailed surgical history for each recruited patient, such as techniques used, age at surgery, bone grafting (if performed), and presence of oronasal fistula (if any) were also collected.

Patients with non-syndromic UCLP were included. Cases with a Simonart's band were eligible if the band measured less than 5 mm in width. Patients with incomplete or isolated cleft lip or palate were excluded.

Prior to data entry, designated personnel from each centre underwent rigorous training and were calibrated to ensure consistency in data collection. The data collection forms were pilot tested prior to study initiation. Each centre was required to submit records for at least 10 patients in each of the following age-based groups: Group I: 5 years ± 6 months, Group II: 12 years ± 6 months, and Group III: 20 years ± 6 months.

All Statistical analyses were undertaken using Stata v18. Frequency distribution and percentages was computed for all the categorical variables while associations between these variables were evaluated using Fisher's exact test. P-value <0.05 was considered to be statistically significant.

## Results

### 5-Year-Olds

A total of 76 patients aged five years were included in the analysis. Eighteen patients (23.7%) received pre-surgical orthopaedic (PSO) treatment using plate appliances, of whom 12 (66.7%) used extraoral strapping and 4 (22.2%) did not have any strapping. Data on strapping were unavailable for the remaining two patients. In the remaining 58 patients (76.3%), the appliance was either not used or information regarding usage was not recorded. Among those who received PSO, three patients (16.7%) continued appliance use up to the time of palatal repair.

Information on the sequence of surgical procedures was available for 56 patients. Of these, 45 patients underwent a staged protocol involving primary lip repair followed by a combined repair of the hard and soft palate as a second intervention. A two-stage approach involving lip and anterior palate repair as the first surgery, followed by hard and soft palate closure as the second intervention, was undertaken in 11 patients. Both lip and palate surgical procedures were most commonly performed before the age of one year, with the mean age at lip repair being 0.45 ± 0.86 years and at palatal closure 0.85 ± 0.92 years.

Information regarding primary lip repair was available for 54 patients (71.05%). Among these, 28 (51.85%) were operated by oral and maxillofacial surgeons and 26 (48.10%) by plastic surgeons. The majority of procedures were performed by surgeons with more than two years of experience (n = 49; 90.74%). Primary rhinoplasty was predominantly undertaken without nasal conformers (n = 47; 87.04%). In the few cases where conformers were used, they were typically fitted one week postoperatively (n = 5; 71.43%).

Separate muscle layer dissection was documented in 33 patients (61.11%), while a vomerine flap was used in 11 patients (20.37%). No postoperative complications were recorded in 48 patients (88.89%), although data were missing for five patients (9.26%).

Millard's rotation-advancement technique was the most frequently used approach for lip repair (n = 41; 75.93%), followed by the Afroze technique (n = 4; 7.41%). The choice of primary lip repair technique did not demonstrate a statistically significant association with the need for secondary lip or nasal revision, secondary velopharyngeal surgery, or fistula formation (p > 0.05; Table 1).

**Table 1.** Effect of Pre-Surgical Orthopedic Treatment and Factors Associated with Primary Lip Repair on Secondary Surgical Procedures in 5-Year-Old.

		Secondary Surgical Procedure								
		Lip Revision			Nasal Revision			Velopharyngeal Surgery		
		Yes n (%)	95% CI	Total	Yes n (%)	95% CI	Total	Yes n (%)	95% CI	Total
Fitting of Presurgical Orthopedic Plate	Yes	1 (5.6)	0.1-27.3	18						
	No	7 (12.7)	5.3-24.5	55						
	Not Recorded	0 (0)	-	3						
Use of Nasal Conformers	Yes	1 (14.3)	0.4-57.9	7	1 (14.3)	7				
	No	5 (10.6)	3.5-23.1	47	1 (2.1)	47				
Primary Rhinoplasty Technique	Closed rhinoplasty	3 (21.4)	4.6-50.7	14	2 (14.3)	1.8-42.8	14			
	Semi-open rhinoplasty	3 (7.9)	1.6-21.4	38	0 (0)	-	38			
	Open rhinoplasty	0 (0)	-	-	0 (0)	-	-			
	Others	0 (0)	-	-	0 (0)	-	-			
Lip Repair Technique	Millard's	4 (9.8)	2.7-23.1	41						
	Fisher's	1 (50.0)	1.3-98.7	2						
	Triangular	0 (0)		2						
	AFROZE Technique	0 (0)		4						
	Not Unknown	1 (20.0)		5						
Vomerian Flap	Yes							0 (0)	-	11
	No							1 (3.7)	0.0-18.9	27
	Not Recorded							0 (0)		16

n: number of patients, %: percentage

Palatal repair was performed in 52 patients (68.42%), most commonly by oral and maxillofacial surgeons (n = 29; 55.77%). Surgeons with more than two years of experience undertook the procedure in 47 cases (90.38%). Intravelar veloplasty was carried out in 29 patients (55.77%), although procedural details were not documented for 15 cases (28.85%). Subperiosteal palatal dissection was the most frequently reported technique (n = 27; 51.92%), predominantly in conjunction with Langenbeck flaps (n = 18; 34.62%) or Bardach's two-flap technique (n = 16; 30.77%).

Postoperative complications occurred in four patients (7.69%), each with a different manifestation: dehiscence, surgical site discharge, fistula, or incomplete closure. Complication data were unavailable for an additional four patients (7.69%). Fistulae were documented in 13 patients (17.11%), although only four (30.77%) were symptomatic, reporting food impaction, nasal regurgitation, or increased nasal discharge. The hard palate was the most common site affected (n = 5; 38.46%), with fistula sizes ranging from 1 to 4 mm.

The effect of presurgical orthopaedics, lip repair and palate repair on the need for secondary surgical procedures, presence of fistula or fistula location showed a really wide confidence intervals, highlighting the uncertainty in the results. The lack of power restricted the use of statistics to calculate the significance level (Table 2).

**Table 2.** Effect of Factors Associated with Palatal Repair on Need for Secondary Surgical Procedures and Site of Fistula Formation in 5-Year-Old.

		Secondary Surgical Procedure											
		Lip Revision			Nasal Revision			Velopharyngeal Surgery			Fistula Closure		
		Yes n (%)	95% CI	Total	Yes n (%)	95% CI	Total	Yes n (%)	95% CI	Total	Yes n (%)	95% CI	Total
Operating Surgeon's Speciality	OMFS	4 (17.4)	4.9-38.7	23	2 (8.7)	1.0-28.0	23	1 (4.3)	0.1-21.9	23	3 (13)	2.7-33.5	23
	Plastic Surgeon	2 (6.9)	0.8-22.7	29	0 (0)	-	29	0 (0)	-	29	2 (6.9)	0.8-22.7	29
Surgeon's Experience	≥ 2 years	5 (10.6)	3.5-23.1	47	1 (2.1)	0.0-11.2	47	0 (0)	-	47	4 (8.5)	2.3-20.3	47
	≤ 2 years	1 (33.3)	0.8-90.5	3	1 (33.3)	0.8-90.5	3	1 (33.3)	0.8-90.5	3	1 (33.3)	0.8-90.5	3
	Mission Surgeon	0 (0)	-	1	0 (0)	-	1	0 (0)	-	1	0 (0)	-	1
	Not Known	0 (0)	-	1	0 (0)	-	1	0 (0)	-	1	0 (0)	-	1
		Site of Fistula											
		Soft/ Hard Palate	Hard Palate										
Palatal Dissection	Supraperiosteal	0 (0)	-	2				1 (4)	0.1-20.3	25	4 (16)	4.5-36.0	25
	Subperiosteal	1 (50)	1.2-98.7	2				0 (0)	-	27	1 (3.7)	0.0-18.9	27
	Langenbeck							1 (5.6)	0.1-27.3	18	2 (11.1)	1.3-34.7	18
	Wardill-Kilner							0 (0)	-	7	0 (0)	-	7
	Vomerine							0 (0)	-	4	0 (0)	-	4
	Bardach (2 flap)							0 (0)	-	16	3 (18.8)	4.0-45.6	16
	Delaire							0 (0)	-	2	0 (0)	-	2
	Others							0 (0)	-	4	0 (0)	-	4
	No flap							0 (0)	-	1	0 (0)	-	1

n: number of patients, %: percentage, OMFS: Oral and Maxillofacial Surgeon.

### For 12-Year-old

A total of 165 patients aged twelve years were included in this analysis. Of these, five patients (3.03%) had received pre-surgical orthopaedic (PSO) appliances, with two (40.0%) also using extra-oral strapping. In the remaining cases, PSO appliance use was either not undertaken or not documented.

Details on the surgical sequence were available for 105 patients. The most frequently reported protocol was a staged approach consisting of primary lip repair followed by combined hard and soft palate closure (n = 59; 56.19%), with the mean age at lip repair being 0.79 ± 1.61 years. A two-stage approach involving lip and anterior palate repair as the first surgery, followed by hard and soft palate closure as the second intervention, was undertaken in 11 patients (10.48%). A three-stage protocol beginning with lip adhesion, followed by definitive lip repair and subsequent palatal surgery, was also recorded in 11 patients (10.48%). Additionally, lip repair combined with soft-palate closure as the initial procedure, followed by hard-palate closure as the second procedure, was performed in 10 patients (9.52%).

Among the 12 year old age group, primary lip repair surgical information was documented in 94 patients (56.97%). Most procedures were performed by plastic surgeons (n = 71; 75.53%), followed by oral and maxillofacial surgeons (n = 18; 19.15%), with the majority of surgeons having more than two years of experience (n = 74; 78.72%).

Primary rhinoplasty was infrequently undertaken in this cohort, with 76.6% of respondents (n = 72) reporting no surgical nasal intervention. The use of nasal conformers was exceptionally uncommon, documented in only one patient (1.1%).

Separate muscle layer dissection was recorded in 35 patients (37.23%), and a vomerine flap was utilised in 11 patients (11.70%). No postoperative complications were reported in 88 patients (93.62%). Millard's rotation-advancement technique was the most commonly employed method for lip repair (n = 64; 68.09%), followed by the triangular flap technique (n = 15; 15.96%).

There was no statistically significant association between the primary lip repair technique and subsequent outcomes, including the requirement for secondary lip or nasal revision, velopharyngeal surgery, or the presence of an oro-nasal fistula (p > 0.05; Table 3).

**Table 3.** Effect of Pre-Surgical Orthopedic Treatment and Factors Associated with Primary Lip Repair on Secondary Surgical Procedures in 12-Year-Old.

		Secondary Surgical Procedure								
		Lip Revision			Nasal Revision			Velopharyngeal Surgery		
		Yes n (%)	95% CI	Total	Yes n (%)	95% CI	Total	Yes n (%)	95% CI	Total
Fitting of Presurgical Orthopedic Plate	Yes	1 (33.3)	0.8-90.5	3						
	No	18 (13.7)	8.4-20.8	131						
	Not Recorded	0 (0)	-	17						
Use of Nasal Conformers	Yes	1 (100)	-	1	1 (100)	-	1			
	No	13 (14.1)	7.7-22.9	92	5 (5.4)	1.7-12.2	92			
Primary Rhinoplasty Technique	Closed rhinoplasty	1 (5.3)	0.1-26.0	19	0 (0)	-	19			
	Semi-open rhinoplasty	13 (18.1)	9.9-28.8	72	6 (8.3)	3.1-17.2	72			
	Open rhinoplasty	0 (0)	-	-	0 (0)	-	-			
	Others	0 (0)	-	-	0 (0)	-	-			
Lip Repair Technique	Millard's	12 (18.8)	10.1-30.4	64						
	Fisher's	0 (0)	-	1						
	Triangular	2 (13.3)	1.6-40.4	15						
	AFROZE Technique	0 (0)	-	8						
	Not Unknown	0 (0)	-	5						
Vomerian Flap	Yes							1 (9.1)	0.2-41.2	11
	No							0 (0)	-	32
	Not Recorded							4 (8.0)	2.2-19.2	50

n: number of patients, %: percentage.

Palatal repair surgical information was available for 79 patients (47.88%). The surgery was predominantly carried out by plastic surgeons (n = 59; 74.68%), with the vast majority having more than two years of surgical experience (n = 67; 84.81%). Intravelar veloplasty was documented in 34 patients (43.04%). However, procedural details were unavailable for 39 patients (49.37%). Supraperiosteal palatal dissection was the most commonly reported technique (n = 42; 53.16%), and the Langenbeck flap (n = 25; 31.65%) and Bardach two-flap technique (n = 38; 48.10%) were the most frequently utilised flap designs.

Regardless of the technique employed, fistula formation or the requirement for repeat palatal repair was noted in 10 patients (12.66%). Overall, oro-nasal fistulae were present in 23 patients (13.94%), with more than half reporting associated symptoms such as nasal discharge, nasal regurgitation of liquids, or food impaction (n = 12; 52.17%). The hard palate was the most commonly affected site (n = 12; 52.17%), and the recorded fistula size ranged from 1 mm to 10 mm.

The effect of primary surgeries and presurgical infant orthopedics on the need for secondary surgical procedures, presence of fistula or fistula location showed wide confidence intervals in the results, undermining the overall precision of the results. (Table 4).

**Table 4.** Effect of Factors Associated with Palatal Repair on Need for Secondary Surgical Procedures in 12-Year-Old.

		Secondary Surgical Procedure											
		Lip Revision			Nasal Revision			Velopharyngeal Surgery			Fistula Closure		
		Yes n (%)	95% CI	Total	Yes n (%)	95% CI	Total	Yes n (%)	95% CI	Total	Yes n (%)	95% CI	Total
Operating Surgeon's Speciality	OMFS	4 (25.0)	7.2-52.3	16	0 (0)	-	16	0 (0)	-	16	0 (0)	-	16
	Plastic Surgeon	8 (13.6)	6.0-24.9	59	5 (8.5)	2.8-18.6	59	4 (6.8)	1.8-16.4	59	13 (22.4)	12.2-34.7	59
	Paediatric Surgeon	0 (0)	-	1	0 (0)	-	1	0 (0)	-	1	0 (0)	-	1
	Not Known	0 (0)	-	3	0 (0)	-	3	0 (0)	-	3	0 (0)	-	3
Surgeon's Experience	≥ 2 years	11 (16.4)	8.4-27.4	67	5 (7.5)	2.4-16.5	67	3 (4.5)	0.9-12.5	67	10 (15.2)	7.5-26.1	66
	≤ 2 years	1 (33.3)	0.8-90.5	3	0 (0)	-	3	1 (33.3)	0.8-90.5	3	2 (66.7)	9.4-99.1	3
	Not Known	0 (0)	-	8	0 (0)	-	8	0 (0)		8	1 (12.5)	0.3-52.6	8
Palatal Dissection	Supraperiosteal							1 (2.4)	0.0-12.5	42	10 (23.8)	12.1-39.4	42
	Subperiosteal							3 (8.1)	1.7-21.9	37	3 (8.3)	1.7-21.9	37

	Langenbeck							1 (4)	0.1-20.4	25	4 (16.7)	4.7-37.4	24
	Wardill-Kilner							0 (0)	-	1	0 (0)	-	1
	Vomerine							1 (33.3)	0.8-90.5	3	1 (33.3)	0.8-90.5	3
	Bardach (2 flap)							1 (2.6)	0.0-13.8	38	7 (17.4)	7.5-33.5	39
	Delaire							0 (0)	-	2	0 (0)	-	2
	Others							1 (10)	0.2-44.5	10	1 (10)	0.2-44.5	10

n: number of patients, %: percentage, OMFS: Oral and Maxillofacial Surgeon.

### For 20 Year old

A total of 66 patients aged 20 years were reviewed. Of these, only two patients (3.03%) had received a presurgical orthopedic plate. Three distinct surgical sequences were identified for lip and palate repair in this age group. An initial lip repair followed by hard and soft palate repair was the most common surgical sequence. The ages for lip and palate surgeries were not reported for 51 patients (77.27%) and 63 patients (95.45%), respectively. In the remaining small cohort, the mean age for lip surgery was at 1.53 ± 2.72 years, and for palate repair was 2.33 ± 1.52 years.

Details of primary lip repair were available for 21 patients (31.82%). Most procedures were performed by plastic surgeons (n = 11; 52.38%), and all surgeons had a minimum of two years of experience. Primary rhinoplasty was undertaken in 21 patients (31.82%), although nasal conformers were used in only one case (4.76%). Muscle dissection as separate layers was performed in six patients (28.57%), and a vomerine flap was utilized in four patients (19.05%). No complications were reported for any of these procedures. Millard's technique was the most commonly used method (n = 9; 42.86%), with the remaining techniques showing relatively even distribution. There was no statistically significant association between the lip repair technique and the requirement for secondary surgical procedures (p > 0.05; Table 5).

**Table 5.** Effect of Pre-Surgical Orthopedic Treatment and Factors Associated with Primary Lip Repair on Secondary Surgical Procedures in 20-Year-Old.

		Secondary Surgical Procedure									
		Lip Revision			Nasal Revision			Velopharyngeal Surgery		
		Yes n (%)	95% CI	Total	Yes n (%)	95% CI	Total	Yes n (%)	95% CI	Total
Fitting of Presurgical Orthopedic Plate	Yes	0 (0)	-	2						
	No	13 (22.4)	12.5-35.2	58						
	Not Recorded	2 (33.3)	4.3-77.7	6						
Use of Nasal Conformers	Yes	1 (100)	-	1	1 (100)	-	1			
	No	5 (25.0)	8.6-49.1	20	2 (10.0)	1.2-31.6	20			
Primary Rhinoplasty Technique	Closed rhinoplasty	0 (0)	-	2	0 (0)	-	2			
	Semi-open rhinoplasty	4 (25.0)	7.2-52.3	16	3 (18.8)	4.0-45.6	16			
	Open rhinoplasty	2 (100)	-	2	0 (0)	-	2			
	Others	0 (0)	-	-	0 (0)	-	-			
Lip Repair Technique	Millard's	4 (44.4)	13.6-78.7	9						
	Fisher's	0 (0)	-	2						
	Triangular	0 (0)	-	1						
	AFROZE Technique	0 (0)	-	4						
	Not Unknown	2 (40)	5.2-85.3	5						
Vomerian Flap	Yes							0 (0)	-	4
	No							0 (0)	-	3
	Not Recorded							2 (14.3)	1.7-42.8	14

n: number of patients, %: percentage.

Primary palatal repair was documented in 19 patients (28.79%), with procedures performed by plastic surgeons in nine cases (47.37%) and oral and maxillofacial surgeons in seven cases (36.84%). Intravelar veloplasty was undertaken in 11 patients (57.89%), and subperiosteal palatal dissection was the most frequently employed approach (n = 13; 68.42%). The Bardach two-flap technique was the most common flap design (n = 8; 42.11%). Postoperative complications were minimal, with 17 patients (89.47%) reporting no issues. Fistula formation was absent in 57 patients (86.36%). Among patients with fistulae, the hard palate was the most frequently affected site (n = 5; 55.56%), with defect sizes ranging from 0.5 to 6 mm.

The results from statistical analysis on the need for secondary surgical procedures, presence of fistula or fistula location showed a really wide confidence intervals, highlighting the uncertainty in the results. (Table 6).

**Table 6.** Effect of Factors Associated with Palatal Repair on Need for Secondary Surgical Procedures in 20-Year-Old.

		Secondary Surgical Procedure											
		Lip Revision			Nasal Revision			Velopharyngeal Surgery			Fistula Closure		
		Yes n (%)	95% CI	Total	Yes n (%)	95% CI	Total	Yes n (%)	95% CI	Total	Yes n (%)	95% CI	Total
Operating Surgeon's Speciality	OMFS	1 (14.3)	0.4-57.8	7	0 (0)	-	7	0 (0)	-	7	1 (14.3)	0.3-57.8	7
	Plastic Surgeon	3 (33.3)	7.4-70.0	9	2 (22.2)	2.8-60.0	9	1 (11.1)	0.3-48.2	9	3 (33.3)	7.4-70.0	9
	Pediatric Surgeon	0 (0)	-	3	1 (33.3)	0.8-90.5	3	1 (33.3)	0.8-90.5	3	2 (66.7)	9.4-99.1	3
Surgeon's Experience	≥ 2 years	4 (21.1)	6.0-45.5	19	3 (15.8)	3.3-39.5	19	2 (10.5)	1.3-33.1	19	6 (31.6)	12.5-56.5	19
	≤ 2 years	0 (0)	-	-	0 (0)	-	-	0 (0)	-	-	0 (0)	-	-
Palatal Dissection	Supraperiosteal							0 (0)	-	6	3 (50.0)	11.8-88.1	6
	Subperiosteal							2 (15.4)	1.9-45.4	13	3 (23.1)	5.0-53.8	13

	Langenbeck							0 (0)	-	-	1 (100)	-	1
	Wardill-Kilner							0 (0)	-	-	0 (0)	-	-
	Vomerine							0 (0)	-	-	1 (33.3)	0.8-90.5	3
	Bardach (2 flap)							0 (0)	-	-	1 (12.5)	0.3-52.6	8
	Delaire							0 (0)	-	-	0 (0)	-	-
	Others							0 (0)	-	-	1 (33.3)	0.8-90.5	3
	No Flap							0 (0)	-	-	1 (100)	-	1

n: number of patients, %: percentage, OMFS: Oral and Maxillofacial Surgeon.

## Discussion

The present study found no significant association between the use of a presurgical orthopedic plate and the need for lip revision surgery. Across all three age groups, a higher proportion of patients did not undergo lip revision surgery regardless of whether a presurgical orthopedic plate had been used.^13,14^ While the presurgical orthopaedic treatment is gaining more popularity and its use is largely increasing among cleft centres, there is substantial variation in clinical protocols and is often influenced by institutional practices and surgeon preference, contributing to ongoing debate regarding their standardized use.^12,13^ Additionally, the evidence on their use from long term studies are also lacking.^14^ A similar pattern was observed in relation to the use of nasal conformers and the subsequent need for lip and/or nasal revision surgeries, with both variables showing no statistically significant associations and a high proportion of patients not requiring secondary revisions. Our observations are consistent with those reported by Garland *et al*., who emphasized the difficulty in drawing definitive conclusions about the long-term benefits of presurgical orthopedic interventions, largely due to heterogeneity in patient management strategies.^15^

There was considerable variation in the type of lip repair technique employed between centres, with no statistically significant association between the specific technique used and the subsequent need for revision surgeries across any age group. The choice of technique is frequently influenced by the surgeon's training, experience, and individual preference.^16,17^ The rate of lip revision surgeries is found to have a heterogeneity. Contrasting the reported increase in secondary revision surgeries, we found that most patients reviewed in our study did not require these revision surgeries, irrespective of the primary lip repair technique used.^6,18^ Among the different lip revision surgical techniques, Millard's rotation-advancement method emerged as the most commonly adopted, consistent with previous reports in the literature.^16,17^ The results of the present study are in accordance with findings of Americleft data that reported no statistically significant association despite the variation in need for revision surgeries.^19^ These variations may reflect differences in surgical expertise, case complexity, or the presence of additional anomalies.

The present study was limited in statistical power due to the small sample size, which restricted our ability to detect the effect of primary surgeries on secondary revision procedures. The wide confidence intervals observed in the data reflect the uncertainty of the estimates and precluded formal statistical significance testing. Therefore, these findings should be considered exploratory and hypothesis-generating rather than definitive. This limitation is consistent with other cleft audits, where retrospective data similarly constrained the ability to draw firm conclusions.^19,20^

The surgeon's preferred technique for either lip or palate repair, their specialty, or their level of experience, showed a statistically significant association with the need for lip or nasal revision surgery. These factors also lacked significant association with the need for secondary velopharyngeal surgery or fistula closure. This trend was consistent across all age groups evaluated in the study. Surgeons from varying specialties, including plastic surgery and oral and maxillofacial surgery, as well as those with differing years of clinical experience, demonstrated comparable outcomes with respect to secondary revision procedures. These findings may suggest that revision surgery outcomes may be more strongly influenced by patient-specific factors, such as the severity and morphology of the cleft, rather than by the surgeon's specialty or seniority. However, drawing this conclusion with wide confidence intervals remains controversial. It is important to point out the sample obtained in the present study may not be large enough to account for the variation in the surgical techniques followed and the number of surgeons involved.

The presence of fistula incidence varies widely, ranging from 0–60%. This can be influenced by several factors, including the surgeon's experience, preferred technique, extent of the cleft, timing of surgery, concomitant presence of cleft lip, and any associated syndromes.^22,25^ While some studies suggest that fistula formation following primary repair can be predicted based on the surgical technique employed, this remains a matter of debate.^21–24^ Importantly, it is universally agreed that a treatment failure rate of approximately 10% is generally not considered high, further underscoring the complexity of care rather than a deficiency in surgical outcomes

Given the variability in cleft presentation, no single surgical technique fits all cases, and the final choice rests with the operating surgeon based on intraoperative findings. Despite these variations, most techniques have yielded favourable results, as reflected in this study. Although data in the CCIS was prospectively collected, certain details were frequently missing from submissions by participating centres, a limitation also noted in the Eurocleft and Americleft projects.^8,26^ As seen in those initiatives, achieving complete data from all centres is challenging. By compiling data from centres across different regions of India, the CCIS has highlighted heterogeneity, which may, in turn, reflect underlying region-specific barriers to cleft care.^27,28^ This analysis extends current evidence and highlights the need for a customised national cleft database capable of capturing regional variations in care delivery and outcomes.

The present study shows that there is no common protocol followed by centres across India. Currently, the choice of surgical care is largely provider-driven. However, there is a growing need for an evidence-based, outcome-driven approach that empowers clinicians to make informed decisions based on the comparative advantages and limitations of each protocol.^29^

## Limitations

A key limitation of this study is its reliance on retrospectively collected data from patient records for primary surgeries. While the majority of patients underwent surgery at their designated treatment centers, some data were missing, primarily due to patients having received their primary surgery at different institutions. Due to the limited statistical power of our sample, we opted to present the data using confidence intervals rather than statistical significance tests for each outcome. Additionally, the sample sizes for the 5 and 20 year old groups were small, reflecting the cleft review protocol in Indian cleft centres, where the first multidisciplinary review typically occurs around seven years of age. Furthermore, 20 year old patients are usually discharged before this age unless they are undergoing extended orthognathic treatment.

## Conclusion

The most common surgical sequence reported was staged lip repair followed by hard and soft palate repair. There was no statistically significant association between the use of a presurgical orthopedic plate and the need for lip revision surgery. No statistically significant association was identified between the lip or palatal surgical technique used and the subsequent need for revision surgeries in any age group assessed. Reported fistula incidence demonstrates wide variability across studies.

Although surgeon specialty, experience level, and the surgical sequence employed did not demonstrate any statistically significant effect on primary surgical outcomes or the need for secondary interventions, these results should be interpreted with caution as they may not fully reflect real-world clinical variation. Further national-level audits comparing outcomes between centres are required to ensure consistent delivery of high-quality, evidence-based cleft care across the country.

## References

[1] Weidler EM Britto MT Sitzman TJ . Facilitators and barriers to implementing standardized outcome measurement for children with cleft lip and palate. Cleft Palate Craniofac J. 2020;58(1):7-18. doi:10.1177/1055665620940187 PMC8155697 32662298

[2] Standards of Approval for Team Care - ACPA, https://acpacares.org/standards-of-approval-for-team-care/ (accessed 11 April 2025).

[3] Shaw W . Global strategies to reduce the health care burden of craniofacial anomalies: Report of WHO meetings on international collaborative research on craniofacial anomalies. Cleft Palate Craniofac J. 2004;41(3):238‐243. doi:10.1597/03-214.1 15151440

[4] Salari N Darvishi N Heydari M Bokaee S , Darvishi F, Mohammadi M. Global prevalence of cleft palate, cleft lip and cleft palate and lip: A comprehensive systematic review and meta-analysis. J Stomatol Oral Maxillofac Surg. 2022;123(2):110‐120. doi:10.1016/j.jormas.2021.05.008 34033944

[5] Putri FA Pattamatta M Anita SES Maulina T . The global occurrences of cleft lip and palate in pediatric patients and their association with demographic factors: A narrative review. Children. 2024;11(3):322. doi:10.3390/children11030322 PMC10969537 38539356

[6] Sitzman TJ Mara CA Long RE Daskalogiannakis J Russell KA Mercado AM Hathaway RR Carle AC Semb G Shaw WC . The americleft project: Burden of care from secondary surgery. Plast Reconstr Surg Glob Open. 2015;3(7):e442. doi:10.1097/GOX.0000000000000415 PMC4527616 26301131

[7] Shaw WC Semb G Nelson P Brattström V Mølsted K Prahl-Andersen B Gundlach KKH . The Eurocleft project 1996–2000: Overview. Journal of Cranio-Maxillofacial Surgery. 2001;29(3):131‐140. doi:10.1054/jcms.2001.0217 11465251

[8] Semb G Brattström V Mølsted K Prahl-Andersen B Shaw WC . The Eurocleft study: Intercenter study of treatment outcome in patients with complete cleft lip and palate. Part 1: Introduction and treatment experience. Cleft Palate Craniofac J. 2005;42(1):64–68. doi:10.1597/02-119.1.1 15643917

[9] O’Brien D Mitra M Jadly M Auslander A Sengupta A . Preoperative nutrition intervention program increases cleft surgery eligibility in the low- and middle-income country setting. BMC Nutr. 2025;11(1):1‐11. doi:10.1186/S40795-025-01031-3/TABLES/5 PMC11877742 40038833

[10] Singh SK . Smile train: The ascendancy of cleft care in India. Indian J Plast Surg. 2009;42(Suppl 1):S192‐S198. doi:10.4103/0970-0358.57186 PMC2825070 19884676

[11] Batra P, Sybil D, Izhar A, Batra P, Thiruvenkatachari B. Standard of care for patients with cleft lip and palate in India-A questionnaire-based study. Cleft Palate Craniofac J. 2023;60(5):536‐543. doi:10.1177/10556656221074212 35099313

[12] Alzain I, Batwa W, Cash A, Murshid ZA. Presurgical cleft lip and palate orthopedics: An overview. Clin Cosmet Investig Dent. 2017;9:53-59. doi:10.2147/CCIDE.S129598 PMC5459959 28615974

[13] Hoshal SG, Solis RN, Tollefson TT. Controversies in Cleft Rhinoplasty. Facial Plast Surg. 2020;36(1):102‐111. doi:10.1055/s-0040-1701477 32191966

[14] Thiruvenkatachari B Chakkaravarthi S Bhuvaraghan A . Effectiveness of nasoalveolar molding treatment for babies with nonsyndromic complete unilateral cleft lip, alveolus, and palate: A systematic review and meta-analysis. Am J Orthod Dentofacial Orthop. 2025;168(1):2‐13.e4. doi:10.1016/j.ajodo.2025.01.012 PMC7617680 40117424

[15] Garland K, McNeely B, Dubois L, Matic D. Systematic review of the long-term effects of presurgical orthopedic devices on patient outcomes. Cleft Palate-Craniofacial Journal. 2022;59(2):156-165. doi:10.1177/1055665621998176/SUPPL_FILE/SJ-PDF-1-CPC-10.1177_1055665621998176.PDF PMC8750140 33678051

[16] Bustamante A, Diaz J, Jones E, Gomez LH, Celie KB, Nuñez J, Auslander A, Magee WP, De Pawlikowski W. Surgical Techniques for Cleft Lip Repair: Insights From Latin America and the Caribbean. Ann Plast Surg. 2025;95(2):125-131. doi:10.1097/SAP.0000000000004379 PMC12278742 40434051

[17] Paradowska-Stolarz A Mikulewicz M Duś-Ilnicka I . Current concepts and challenges in the treatment of cleft lip and palate patients—A comprehensive review. J Pers Med. 2022;12(12):2089. doi:10.3390/jpm12122089 PMC9783897 36556309

[18] Sitzman TJ Coyne SM Britto MT . The burden of care for children with unilateral cleft lip: A systematic review of revision surgery. Cleft Palate-Craniofacial Journal. 2016;53(4):84‐94. doi:10.1597/14-202 26247708

[19] Sitzman TJ, Carle AC, Lundberg JN, Heaton PC, Helmrath MA, Trotman CA, Britto MT. Marked variation exists among surgeons and hospitals in the use of secondary cleft lip surgery. Cleft Palate Craniofac J. 2019;57(2):198. doi:10.1177/1055665619880056 PMC6957675 31597471

[20] Ng JJ, Banala M, Massenburg BB, Romeo DJ, Wu M, Jackson OA, Low DW, Bartlett SP, Swanson JW, Taylor JA. Long-Term surgical outcomes of primary rhinoplasty. Cleft Palate Craniofacial Journal. 2025;62:1733‐1741. doi: 10.1177/10556656241272736 39262157

[21] Cohen SR, Kalinowski J, LaRossa D, Randall P. Cleft palate fistulas: A multivariate statistical analysis of prevalence, etiology, and surgical management. Plast Reconstr Surg. 1991;87(6):1041‐1047. http://www.ncbi.nlm.nih.gov/pubmed/2034725 2034725

[22] Amaratunga NA de S. Occurrence of oronasal fistulas in operated cleft palate patients. J Oral Maxillofac Surg. 1988;46(10):834‐837. doi:10.1016/0278-2391(88)90044-4 3171742

[23] Losken HW, Van Aalst JA, Teotia SS, Dean SB, Hultman S, Uhrich KS. Achieving low cleft palate fistula rates: Surgical results and techniques. Cleft Palate-Craniofacial Journal. 2011;48(3):312‐320. doi:10.1597/08-288 20815713

[24] Timbang MR, Gharb BB, Rampazzo A, Papay F, Zins J, Doumit G. A systematic review comparing furlow double-opposing Z-plasty and straight-line intravelar veloplasty methods of cleft palate repair. Plast Reconstr Surg. 2014;134(5):1014‐1022. doi:10.1097/PRS.0000000000000637 25347635

[25] Park MS Seo HJ Bae YC . Incidence of fistula after primary cleft palate repair: A 25-year assessment of one surgeon’s experience. Arch Plast Surg. 2022;49(1):43. doi:10.5999/aps.2021.01396 PMC8795648 35086308

[26] Long RE, Hathaway R, Daskalogiannakis J, Mercado A, Russell K, Cohen M, Semb G, Shaw W. The Americleft study: An inter-center study of treatment outcomes for patients with unilateral cleft lip and palate part 1. Principles and study design. Cleft Palate Craniofac J. 2011;48(3):239‐243. doi:10.1597/09-180.1 21219224

[27] Weatherley-White RCA, Eiserman W, Beddoe M, Vanderberg R. Perceptions, expectations, and reactions to cleft lip and palate surgery in native populations: A pilot study in rural India. Cleft Palate-Craniofacial Journal. 2005;42(5):560‐564. doi:10.1597/04-003.1 16149840

[28] Naram A, Makhijani SN, Naram D, Reddy SG, Reddy RR, Lalikos JF, Chao JD. Perceptions of family members of children with cleft lip and palate in hyderabad, India, and its rural outskirts regarding craniofacial anomalies: A pilot study. Cleft Palate-Craniofacial Journal. 2013;50(3):e41-e46. doi:10.1597/10-170 22519557

[29] Crerand CE, Conrad AL, Albert M, Crilly Bellucci C, Heppner CE, Sheikh F, Woodard S, Kapp-Simon KA. The Americleft psychosocial outcomes project: a multicenter approach to advancing psychosocial outcomes for youth with cleft lip and palate. Clin Pract Pediatr Psychol. 2017;5(1):65. doi:10.1037/CPP0000172 PMC6738562 31511822

